# Effect of Luting Cement and Convergence Angle of the Preparation on the Internal Fit of Zirconia Restorations

**DOI:** 10.3390/ma14247858

**Published:** 2021-12-18

**Authors:** Andrés Sánchez-Monescillo, Carlos González-Serrano, José González-Serrano, João Malta Barbosa, Carlos López-Suárez, Sillas Duarte

**Affiliations:** 1 Norris Dental Science Center, Division of Restorative Sciences, Herman Ostrow School of Dentistry, University of Southern California, 925 W 34th St, DEN 311, Los Angeles, CA 90089, USA; sillas.duarte@usc.edu; 2 IDIBO Research Group, Stomatology Department, School of Health Sciences, Rey Juan Carlos University, Av. de Atenas, S/N, Alcorcón, 28922 Madrid, Spain; gonzalezserrano.carlos@gmail.com; 3 ORALMED Research Group, Department of Dental Clinical Specialities, School of Dentistry, Complutense University of Madrid, Plaza Ramón y Cajal s/n, 28040 Madrid, Spain; jose.gser@gmail.com; 4 Department of Biomaterials and Biomimetics, New York University College of Dentistry, 345 E 24th St, New York, NY 10010, USA; joaomaltabarbosa@gmail.com; 5 ICAPE Research Group, Department of Conservative Dentistry and Buccofacial Prosthesis, School of Dentistry, Complutense University of Madrid, Plaza Ramón y Cajal, s/n, 28040 Madrid, Spain; carlop04@ucm.es

**Keywords:** internal fit, coping, restoration, zirconia, cement, luting cement, preparation angle, preparation design, occlusal convergence

## Abstract

The objective was to evaluate the effect of luting agents and the preparation design on the internal fit of zirconia restorations. Sixty dies were prepared and divided in occlusal convergence angle of 6° (OC6) and 12° (OC12). CAD/CAM zirconia copings were fabricated (Lava All-Ceramic System). A zinc phosphate cement (ZPC); a glass ionomer cement (GIC); and a resin cement (RC) were studied. Specimens were sectioned and coping/die discrepancies were evaluated through Stereoscopic Microscopy. A closer fit was observed in OC12 when compared to OC6 (*p* < 0.001). For OC6 no significant differences were observed in between ZPC, GIC, and RC (*p* > 0.05). For OC12, a significantly closer fit was recorded on the ZPC subgroup when compared to the GIC subgroup (*p* < 0.001). Preparations of 12 degrees demonstrated a closer internal fit when compared to 6 degrees. Preparations of 12 degrees achieved better internal fit values with ZPC (Fortex) followed by RC (RelyX Unicem), and GIC (Ketac Cem). No differences were found when comparing different luting agents over 6° degrees preparations.

## 1. Introduction

Through the years, several definitions have been proposed for the term internal adaptation or internal fit [1,2,3]. From a dental perspective, internal adaptation can be defined as the accuracy with which a restoration sits onto the corresponding tooth preparation [4].

An inadequate internal fit can contribute to microleakage, thus predisposing the abutment tooth to caries, sensitivity, and loss of vitality or even fracture [5,6]. Moreover, the poorer the adaptation between the intaglio surface of the restoration and the tooth preparation, the lower the retention [7,8]; increasing the likelihood of restoration instability and decementation, particularly when the luting agent presents a higher solubility index [9,10].

Several factors can influence the restoration’s internal adjustment, both clinically as well as in the dental laboratory. From a clinical standpoint, several critical factors are dependent upon the clinician’s knowledge and execution capabilities related with tooth preparation design (total preparation occlusal convergence angle, finish line design, occlusal reduction, and surface roughness), impression material selection, and technique, all capable of impacting upon the accuracy of the working cast and, therefore, affecting the resulting restorations. From the dental laboratory perspective, apart from a knowledgeable and careful material selection and execution, adjusting the thickness of cement spacing to meet the future requirements of the luting agent and cementation technique will ultimately be determinant to improve the clinical fit of the restoration after cementation [4,5,9,10,11].

The plethora of the currently available luting agents’ types, brands, and variations poses an increased difficulty for researchers to establish comprehensive comparisons in terms of their clinical and laboratorial performances. Therefore, physical properties of different cements such as viscosity, wettability, stability, and strength may play a crucial role in long term treatment result. For this reason, the clinician should support the selection criteria of the most adequate luting agent for a particular restoration and patient through a case specific evaluation that considers among other factors the abutment substrate, restoration material, esthetic needs, and clinician experience [12].

Dental adhesive systems have improved considerably through the years; however, these improvements still do not result in what could be described as a “material independent performance” due to the limitations related with bonding to zirconia surfaces [12,13,14,15,16,17,18]. In addition, clinical situations where efficient moisture control cannot be attained may result in an underperformance of adhesive materials and techniques. For this reason, water-based luting agents such as zinc phosphate (ZP) and glass ionomer (GI) are still considered to be a valid alternative to adhesive resin cements (RC) for zirconia restorations [12,13].

Therefore, understanding the influence of internal adaptation in the success of fixed prosthodontics is a clinically revenant factor to predict the outcome and longevity of a given restoration. Several studies have shown that factors such as preparation and restoration design, restorative material, manufacturing system, and luting agent, can influence the restoration/abutment fit [3,9,10,11,19,20]. This multitude of influencing factors may help explain the disparity of results reported among studies regarding restoration fit [6,21,22].

Although internal fit of zirconia restorations has been widely investigated, interaction between luting cement and convergence angle of the preparation remains controversial, since both factors may have an impact on the internal fit of zirconia restorations. Thus, the present work aimed to investigate how different luting agents and total occlusal convergence angles of dental preparations influence the internal adaptation of zirconia restorations. 

The null hypothesis was that no significant differences would be seen on the internal adaptation of zirconia copings regardless of the selected luting agent and total occlusal convergence angles of dental preparations.

## 2. Materials and Methods

### 2.1. Abutment Design and Production

**Figure 1:**
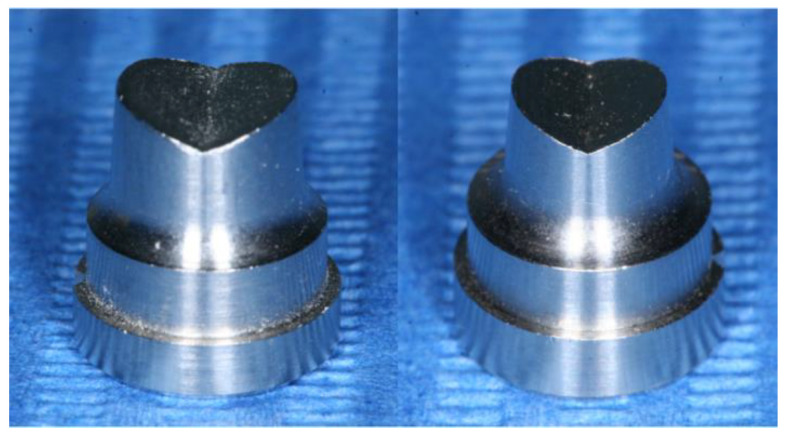
Dies with 6-degree preparation angle (**left**) and 12-degree preparation angle (**right**).

Ninety dies mimicking a mandibular first premolar preparation shape of 6.0 mm in diameter and 6.0 mm in height were designed and milled in stainless steel to be used as abutments for correspondent zirconia copings. The preparation design comprised also an anatomical, V-shaped, occlusal reduction with rounded angles as well as a chamfer marginal preparation with a 1.0 mm depth (Figure 1). Forty-five abutments were produced with a total occlusal convergence angle preparation of 6 degrees (OC6) and the remaining forty-five with a total occlusal convergence angle preparation of 12 degrees (OC12).

### 2.2. Zirconia Copings Design and Production

**Figure 2:**
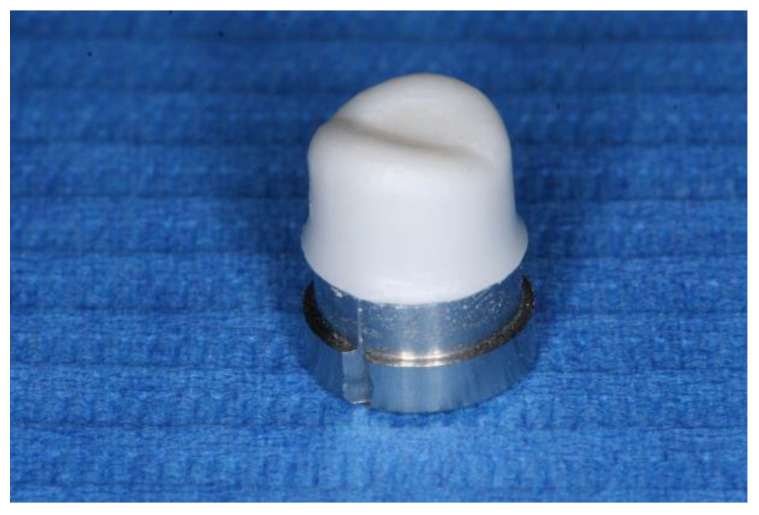
Zirconia coping cemented with a pressure of 20 N with the cement excess removed.

The zirconia copings were designed and produced following a CAD/CAM workflow (Lava All Ceramic System, 3M Oral Care, St Paul, MN, USA). An occlusion spray (White Yeti Dental, YETI Dentalprodukte, Engen, Germany) was applied onto the die preparation surface to eliminate surface gloss resulting from the scanner’s light. On the CAD software, the digital die was identified as a mandibular right first premolar. Coping design parameters consisted of an overall homogenous thickness of 0.5 mm, a 30 μm digital spacing for the cement layer, covering 80% of the die surface and tending to 0 μm at the cervical margin. The resulting design was exported to the CAM module of the software and twenty pre-sintered 40 mm Lava Zirconia Blocks were used to mill the copings. Upon completion of the milling process, finishing and polishing was carried with a slow speed handpiece. Finally, copings were sintered at 1550 °C for 12 h (Lava Furnace 200, 3M Oral Care) (Figure 2).

### 2.3. Coping Cementation

Three luting agents with clinical indication for the cementation of zirconia-based restorations were selected for testing: a hand-mix zinc phosphate cement (ZPC) (Fortex, Faciden), a self-mixing and single-dose capsule glass ionomer cement (GIC) (Ketac Cem Aplicap, 3M Oral Care, St Paul, MN, USA); and an auto-mixture resin cement (RC) (RelyX Unicem 2 Automix, 3M Oral Care, St Paul, MN, USA). The cementation procedures were performed by a single operator, who performed 5 pre-testing sample assemblies per cement group (ZPC, GIC and RC) both in the OC6 and OC12 dies (total pre-testing *n* = 30).

Following the previously described calibration, the testing zirconia copings were cemented over their respective abutments by the same operator calibrated under identical conditions of temperature and humidity. Luting agents were manipulated following the manufacturer recommendations. The mixture was performed for each sample individually and then applied onto the intaglio surface of the zirconia coping. A controlled and constant cementation pressure of 20 N was applied for all samples. This pressure was kept for 3 min for ZPC, 7 min for GIC and 6 min for RC. In summary, after cementation, six groups (*n* = 10) were obtained as follows:

- Group 1: OC6 + ZPC.
- Group 2: OC6 + GIC.
- Group 3: OC6 + RC.
- Group 4: OC12 + ZPC.
- Group 5: OC12 + GIC.
- Group 6: OC12 + RC.

### 2.4. Sample Sectioning

All the specimens were embedded in transparent polyester resin to ensure coping/die stability during sample sectioning. A mechanical precision saw (ISOMET 5000, Buehler, Glenview, IL, USA) with a diamond blade (ISOMET Diamond Wafering Blade 127 × 0.4 mm, Glenview, IL, USA) at 2500 rpm and a disc feed rate of 6 mm per minute under abundant irrigation was used. Sample sectioning was always performed in the middle of the specimen bucco-lingually and mesio-distally following the longitudinal axis.

### 2.5. Evaluation of Internal Fit

The internal fit evaluation of the coping/die assemblies was performed through computer-guided dual screen image analysis microscopical measurement (Stereoscopic Microscope, Leica M80 and Leica LAS V4.0, Leica, Microsystems Ltd., Heerbrugg, Switzerland). The internal surface of each sectioned sample was positioned perpendicularly to the microscope’s tubular axis, maintaining an unaltered working distance and a 1.6x magnification. A photograph was taken with the microscope’s digital camera (Leica DFC450, Leica Microsystems, Wetzlar, Germany), a high quality 5 Megapixel CCD sensor with a resolution of 1280 × 960 pixels.

**Figure 3:**
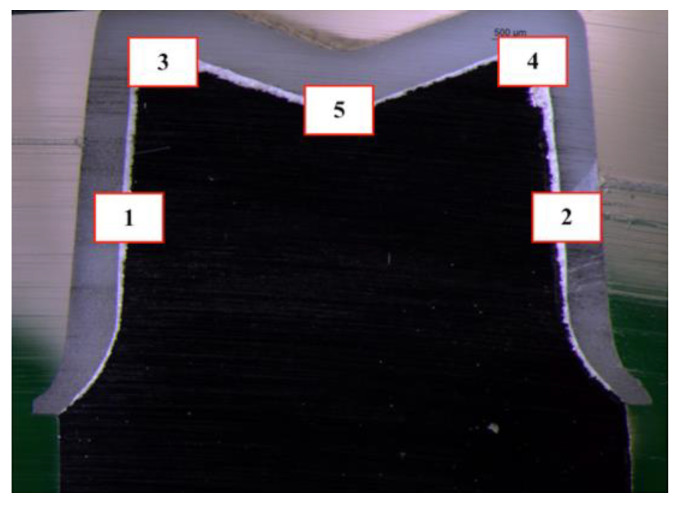
Five areas selected for the measurements using a Stereoscopic Microscope at 1.6× magnification.

**Figure 4:**
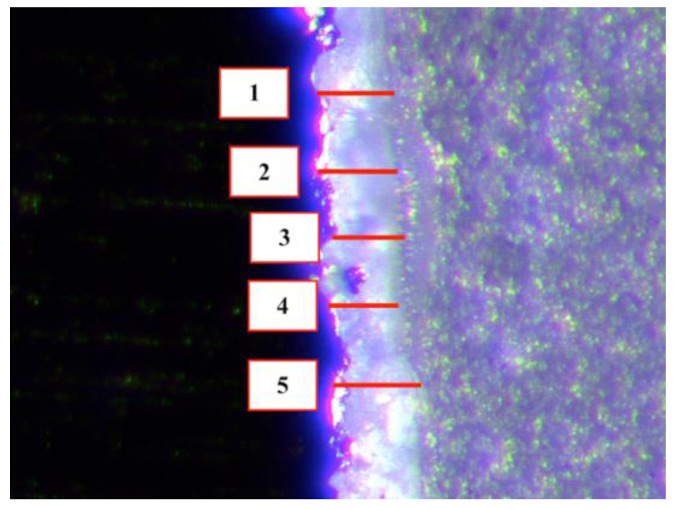
Five equidistant linear measurements using a Stereoscopic Microscope at 40× magnification.

Standardized internal measurements were performed in five specific locations of the complex coping-abutment: left (1) and right (2) axial walls distancing 1.5 mm from the central fossa; left (3) and right (4) cusp tips and pit of the preparation (5) (Figure 3). For each location five equidistant linear measurements were taken under 40× digital image magnification (Figure 4). In total, 25 measurements were made per specimen.

The recorded results were evaluated and compared statistically considering luting agent and total occlusal convergence angle as variables.

### 2.6. Statistical Analysis

A Statistical Package for Social Sciences (SPSS) software (version 2.0, SPSS Inc, Chicago, IL, USA) was used to perform the statistical analysis. Descriptive statistics were obtained. Data were assessed for a normal distribution using the Kolmogorov–Smirnov test. Since the resulting data were not in accordance with the theorem of central data distribution, non-parametric Kruskal–Wallis and Tukey’s tests were performed for statistical evaluation. The results were assessed with 95% confidence interval and a significance level of *p* < 0.05.

## 3. Results

### 3.1. Luting Agents

The average value of internal misfit was 74.99 µm (±54.16) for ZPC, 82.47 µm (±59.20) for GIC, and 80.88 µm (±73.38) for RC (Table 1). There were no statistically significant differences in the comparison of the different luting agents (*p* = 0.088) (Table 1).

### 3.2. Total Occlusal Convergence Angles

The average value of internal misfit was 100.49 µm (±65.78) for 6OC and 58.40 µm (±51.89) for 12OC. A statistically significant reduction was found in the internal fit of 12OC when compared to 6OC (*p* < 0.001) (Table 2).

### 3.3. Luting Agents and Total Occlusal Convergence Angles

6OC presented a mean internal misfit of 104.63 µm (±46.43) with ZPC, 93.94 (±63.59) µm with GIC and 102.91 µm (±82.15) with RC. No statistically significant differences were obtained in the comparison of different luting agents in combination with 6OC (*p* > 0.05) (Table 3).

12OC presented a mean internal misfit of 45.34 µm (±44.23) with ZPC, 71.01 µm (±52.12) with GIC and 58.86 µm (±55.65) with RC (Table 3). For OC12, a statistically significant reduction was observed in the internal fit with ZPC when compared to GIC (*p* < 0.001) (Table 3).

## 4. Discussion

The internal fit of fixed restorations has been the focus of several studies [23,24,25]; however, investigations of the combined influence of different luting agents with various total occlusal convergence angles are still limited regarding this particular factor, whose results have reported mainly on retention, fracture resistance, and marginal fit [9,10,12,26,27,28,29]. Water-based luting agents such as ZPC and GIC seem to contribute to an improved fit when compared to RC [9,10]. This can be explained by the influence of viscosity on film thickness when sitting the restoration over the preparation [9,10,12]. Despite this, Hmaidouck et al. (2011) reported lower internal mismatch values for RC in comparison with ZPC [29]. Such differences can be explained by the amount of luting agent spacing provided by the dental technician as for RC an improved internal fit is expected when 100 μm spacing is provided when compared to 50 μm spacing [9,29].

As for the influence of the abutment preparation angles, several studies have confirmed it to be a crucial factor influencing the restoration’s internal fit [10,11,30,31,32]. Angles between 4 and 10 degrees have been traditionally recommended in order to improve retention. However, as these results are difficult to obtain intra-orally, clinical recommendations range between 10-degree and 20-degree preparation angles [33,34]. This may also result in an improvement of internal fit as a greater preparation total occlusal convergence angle facilitates the seating of the restoration and the escape of the excessive luting agent [9,30,34,35,36,37], even though some studies have reported better restoration fits with 6-degree preparations when compared with 20-degree, leaving this topic still open to some debate [10,33].

What seems to be clear is the combined influence of luting agents and preparation design [9,10,29]. Regarding this, the present study showed that while different luting agents led to similar fit values in 6-degree preparations, significant differences were observed for 12-degree preparations. This seems to indicate that an increase in the preparation angle may overcome the luting agent selection issue.

Even though other variables capable of influencing the post cementation internal fit of zirconia restorations were not reproduced, this study showed that the greater the convergence angle of the preparation, the greater the influence of cement on the internal fit of the restorations. This way, cement selection remains as a crucial decision when it comes to internal fit of ceramic restorations. From a clinical point of view, low viscosity cements such as zinc phosphate-based cements seem to provide better internal values compared to glass-ionomer and resin-based cements. These results may orientate clinicians in their daily practice when cement selection is considered.

Although this study enlightens the complex interaction between cements and preparation design and its influence on the internal fit of ceramic restorations, further studies should be conducted for a better understanding since multiple factors may influence on restoration internal fit.

## 5. Conclusions

This study shows the remarkable relation between preparation design and cements when internal fit of ceramic restorations is evaluated. In this way, interaction between luting cements and convergence angle of the preparation on internal fit of zirconia restorations was demonstrated. Preparations with a total occlusal convergence angle of 12-degrees achieved better internal fit values when compared with 6-degree preparations. Lower internal fit values were found for ZPC, followed by GIC and RC with 12-degree occlusal convergence angle of the preparation. Despite this, luting cements showed no differences in internal fit when combined with a 6-degree occlusal convergence angle of the preparation. It seems clear that higher degrees of convergence in the preparation design increases the internal adaptation of the restorations to the abutment preparation after cementation. ZPC produces better internal fit results compared with other cements such as GIC and RC. Furthermore, the greater convergence angle of the preparation the greater impact of the cement in the internal fit of ceramic restorations.

## Figures and Tables

**Table 1:** Internal fit according to the type of cement used. SD: standard deviation.

Cement Group	*n*	Internal Fit (µm) Mean (SD)	Other Cements	*p*	
Zinc phosphate cement (Fortex)	20	74.99 (54.16)	Glass ionomer cement (Ketac Cem)	0.143	0.088
			Resin cement (RelyX Unicem)	0.299	
Glass ionomer cement (Ketac Cem)	20	82.47 (59.20)	Zinc phosphate cement (Fortex)	0.143	
			Resin cement (RelyX Unicem)	0.915	
Resin cement (RelyX Unicem)	20	80.88 (73.38)	Zinc phosphate cement (Fortex)	0.299	
			Glass ionomer cement (Ketac Cem)	0.915	

**Table 2:** Internal fit according to the preparation angle. SD: standard deviation.

Preparation Angle Group	*n*	Internal Fit (µm) Mean (SD)	*p*
6-degree	30	100.49 (65.78)	<0.001
12-degree	30	58.40 (51.89)	

**Table 3:** Internal fit according to the cement used and the preparation angle. SD: standard deviation.

Cement and Preparation Angle Groups	*n*	Internal Fit (µm)	Other Cements and Preparation Angles	*p*
		Mean (SD)		
Zinc phosphate cement (Fortex) and 6-degree	10	104.63 (46.43)	Glass ionomer cement (Ketac Cem) and 6-degree	0.323
			Resin cement (RelyX Unicem) and 6-degree	1
			Zinc phosphate cement (Fortex) and 12-degree	<0.001
			Glass ionomer cement (Ketac Cem) and 12-degree	<0.001
			Resin cement (RelyX Unicem) and 12-degree	<0.001
Glass ionomer cement (Ketac Cem) and 6-degree	10	93.94 (63.59)	Zinc phosphate cement (Fortex) and 6-degree	0.323
			Resin cement (RelyX Unicem) and 6-degree	0.527
			Zinc phosphate cement (Fortex) and 12-degree	<0.001
			Glass ionomer cement (Ketac Cem) and 12-degree	<0.001
			Resin cement (RelyX Unicem) and 12-degree	<0.001
Resin cement (RelyX Unicem) and 6-degree	10	102.91 (82.15)	Zinc phosphate cement (Fortex) and 6-degree	1
			Glass ionomer cement (Ketac Cem) and 6-degree	0.527
			Zinc phosphate cement (Fortex) and 12-degree	<0.001
			Glass ionomer cement (Ketac Cem) and 12-degree	<0.001
			Resin cement (RelyX Unicem) and 12-degree	<0.001
Zinc phosphate cement (Fortex) and 12-degree	10	45.34 (44.23)	Glass ionomer cement (Ketac Cem) and 12-degree	<0.001
			Resin cement (RelyX Unicem) and 12-degree	0.105
			Zinc phosphate cement (Fortex) and 6-degree	<0.001
			Glass ionomer cement (Ketac Cem) and 6-degree	<0.001
			Resin cement (RelyX Unicem) and 6-degree	<0.001
Glass ionomer cement (Ketac Cem) and 12-degree	10	71.01 (52.12)	Zinc phosphate cement (Fortex) and 12-degree	<0.001
			Resin cement (RelyX Unicem) and 12-degree	0.190
			Zinc phosphate cement (Fortex) and 6-degree	<0.001
			Glass ionomer cement (Ketac Cem) and 6-degree	<0.001
			Resin cement (RelyX Unicem) and 6-degree	<0.001
Resin cement (RelyX Unicem) and 12-degree	10	58.86 (55.65)	Zinc phosphate cement (Fortex) and 12-degree	0.105
			Glass ionomer cement (Ketac Cem) and 12-degree	0.190
			Zinc phosphate cement (Fortex) and 6-degree	<0.001
			Glass ionomer cement (Ketac Cem) and 6-degree	<0.001
			Resin cement (RelyX Unicem) and 6-degree	<0.001

## Data Availability

The study data is saved by Andrés Sánchez-Monescillo.
